# Determinants of rural practice among a cohort of dental professionals in Australia

**DOI:** 10.1186/s12909-025-06681-2

**Published:** 2025-01-29

**Authors:** Santosh Kumar Tadakamadla, Sudheer Babu Balla, Jyothi Tadakamadla, Libby Semmens, Sarah Down, Carol McKinstry, Jane Mills

**Affiliations:** 1 Dentistry and Oral Health, Department of Rural Clinical Science & Violet Vines Marshman Centre for Rural Health Research, La Trobe Rural Health School, La Trobe University, Bendigo, VIC 3550 Australia; 2 La Trobe Rural Health School, La Trobe University, Bendigo, VIC 3550 Australia; 3 Dentistry and Oral Health, Department of Rural Clinical Sciences, La Trobe Rural Health School, La Trobe University, Bendigo, VIC 3550 Australia; 4 Violet Vines Marshman Centre for Rural Health Research, La Trobe Rural Health School, La Trobe University, Bendigo, VIC 3550 Australia

**Keywords:** Dental and oral health students, University, Rural origin, Practice, Workforce, Australia

## Abstract

**Background:**

Most research on tracking practice locations of health students has focused on medical students, particularly the factors influencing their choice to work in rural and remote areas. However, there is limited research on how rural origin and training in regional or rural settings affect the employment destinations of dental and oral health graduates. This paper explores the practice locations of dentistry and oral health therapy (OHT) graduates from rural backgrounds compared to those from metropolitan areas in Australia.

**Materials and methods:**

The target population was dental and OHT graduates from La Trobe University’s Rural Health School (Australia) who completed their studies between 2009 and 2023. The graduates’ primary place of practice was sourced from the Australian Health Practitioners Regulation Agency (AHPRA) data. The 2019 Modified Monash Model (MMM) was used to categorise the students’ original place of residence by rurality and practice locations. Multivariable analyses were conducted to explore the association between home and practice locations while controlling for the effect of socio-demographic characteristics collected from students at enrolment.

**Results:**

Data were available for 819 graduates matched to the AHPRA register. Of these, 541 (66.1%) were dentists, and 278 (33.9%) were OHTs. The majority were female (56.7% dentists and 81.7% OHTs), 11.3% (dentists) and 21.6% (OHTs) of the graduates originated from rural and remote areas, 16.6% (dentists) and 18% (OHTs) from regional areas, and 72.1% (dentists) and 60.4% (OHTs) from metropolitan areas. Multinomial logistic regression analyses for dentists and OHTs identified that , having a regional background, or having a rural or remote background were the most significant predictors for regional, rural/remote practice over metropolitan areas.

**Conclusion:**

Regional background is the strongest predictor for graduate dentists and OHTs practicing in Australia’s regional or rural and remote locations. Similarly, students from rural and remote locations were highly likely to practice in rural/ remote locations. Increasing the recruitment of students with rural backgrounds may positively impact graduates’ decisions to practice in rural areas.

## Background

Approximately 7 million Australians reside in rural and remote regions, facing distinctive challenges due to their geographical isolation [1]. Compared to urban counterparts, rural residents often experience inferior health outcomes [2]. Poor oral health is prevalent, exacerbated by increased remoteness [3, 4]. The causes of this disparity are multifaceted, including difficulties in recruiting and retaining the dental workforce, further limiting access to care [5, 6]. The severity of these disparities calls for immediate attention and action.

The World Health Organisation (WHO) highlighted rural workforce shortages as a substantial barrier to universal and equitable health coverage [7]. In Australia, persistent workforce challenges exist, notably due to pronounced maldistribution of dental practitioners (dentists, dental hygienists, therapists, prosthetists and oral health therapists (OHTs) [8]. Major cities have higher density of dentists (full-time equivalent (FTE) rate of 63.8) and other dental professionals (OHTs of 7.4) per 100,000 population than remote areas (26.3 FTE dentists and 4.4 OHTs) [9, 10]. The National Rural Health Alliance (NRHA) in Australia states that rural communities require an additional 21,357 dental practitioners to reach parity of coverage as in major cities on a per-population basis [11]. Significantly lower utilisation rates of dental services through the Child Dental Benefits Schedule (CDBS) also exist in rural/ remote areas than in major cities, particularly among First Nations children [12]. NRHA recommends addressing the workforce distribution challenges in rural and remote communities for improving oral health status of these children [13].

In Australia, various strategies have been implemented to address the challenge of dental practitioner maldistribution, ranging from using foreign-trained dentists to financial incentives such as student loan repayment programs [14–18]. Research findings indicate that recruiting students of rural origin is the most influential factor to increase the likelihood of them taking up rural practice [18, 19]. However, research examining the factors influencing dental graduates’ decisions regarding their practice location upon graduation is limited. There are two notable studies in the Australian context, however, they were conducted approximately two decades ago and with small samples of dental graduates [16, 20].

Rural immersion is crucial in dental education programs [21]. Some studies involving medical students explored this approach [22, 23]; however, research focusing on students in regional or rural dental education programs is lacking, especially using a large dataset in the Australian context. This study investigates original place of residence by rurality on the practice destination of dentistry and OHT graduates in Australia using the graduate dataset from a dental education provider in Australia.

## Methods

### Participants

The target population for this study consists of graduates who completed a dentistry or oral health therapy program from the La Trobe Rural Health School (La Trobe University) at Bendigo between 2009 and 2023. In Australia, nine education providers currently offer programs that result in registrable qualification as a dentist, while eight organisations offer Oral Health therapy programs. La Trobe Rural Health School, Australia’s largest rural health school, currently provides dentistry and oral health therapy programs. The Bachelor of Oral Health Science course (BOHSc) was introduced at the Bendigo campus in 2005 to address a workforce shortage of oral health therapists in rural Victoria. Some Government funding was received for the necessary infrastructure and other expenses, and a 2.5-year course with a summer semester was established to graduate OHTs as soon as possible to fill vacancies. In 2008, a five-year dentistry course was introduced with additional Government funding to develop state-of-the-art teaching and simulation facilities and student accommodation. Both courses utilise public dental services within regional Victoria for extensive practical clinical experience. Since course commencement, enrolments in the dentistry and OHT course have grown. To increase the percentage of dentistry and OHT students of rural origin, a separate admission entry pathway was added in 2020, which lifted the number of rural students, resulting in 79 per cent of the dentistry cohort being of rural origin in the 2024 intake compared to 9% in 2019.

### Data sources and collection

This study is approved by the La Trobe University Human Research Ethics Committee (HEC24112). A waiver of consent was approved by the La Trobe University Human Research Ethics Committee in accordance with Sect. 2.3.10 (a-i) of the National Statement on Ethical Conduct in Human Research, Australia.

Data were gathered from the university’s internal administrative databases, including the student’s age at course commencement, gender, home address, language spoken at home, and area-level Socioeconomic status (SES), and the locations of their placements during the study. The graduates’ geographical origin was identified using the hometown postcode recorded at their initial enrolment. Data on graduates’ practice location was retrieved from the Australian Health Practitioners Regulation Agency (AHPRA) registry [24]. International students who moved back to their country of origin, those who did not register with AHPRA and those with no home address were excluded. The Modified Monash Model (MMM) classification was employed to categorise the home and practice locations into Metropolitan areas (MM1), regional centres (MM2), large rural towns (MM3), medium rural towns (MM4), small rural towns (MM5), remote communities (MM6), and very remote communities (MM7) [25]. MMM categories 3–7, which represent rural and remote communities, were all grouped into one category in this study due to the limited number of graduates from these areas. All data were manually checked for duplicates and missing entries.

### Placements

Dentistry students at La Trobe Rural Health School undertake clinical placements from the third year of their program. In the third year, all students undertake year-long clinical placements in Bendigo (MM2). Students participate in two rotations of approximately 18 weeks each in the fourth and fifth years. The clinical placements in the 4th and 5th years are predominantly in community dental agencies across rural Victoria. However, one of these was re-classified as metropolitan (MM1) in 2019 when the MMM classification was updated to align with new census data. Students in the OHT course commence their clinical placements in 2nd year, with all placements undertaken in Bendigo (MM2).

### Outcome/dependent variable

The primary outcome of this study is the principal place of practice after graduation. It was divided into three categories: “metropolitan (MM1),” “regional (MM2),” and rural/ remote (MM3- 7),” respectively.

### Independent variables and covariates

The primary independent variable was the graduates’ home postcodes categorised into three groups: metropolitan (MM1), regional (MM2), and rural/remote (MM3-7).

The covariates included in this study were the students’ gender, the age at enrolment, language spoken at home, and SES. Within the university student data collection framework, data on gender is categorised as males and females. The language spoken at home is collected as “English-speaking” or “Non-English speaking”. SES data is collected in three categories: “low,” “medium,” and “high” based on the SES of the statistical area 1 (SA1) of students’ home residence according to the Australian Socio-Economic Index for Areas – Index of Education and Occupation (SEIFA). Those who live in the bottom 25% of SA1 are considered low SES, while those in the middle 50% and top 25% are considered medium and high SES, respectively.

### Statistical analysis

Data management and statistical analysis were performed using IBM SPSS software version 29.0 (SPSS Inc., Chicago, IL, USA). Descriptive statistics were used to describe the sample characteristics. Chi Square analysis was conducted to explore the association between the remoteness of the home location and the practice location after graduation. Multinomial logistic regression analyses were conducted to examine the association of the independent variable and covariates with the outcome variable for dentistry and OHT graduates. First, a series of unadjusted analyses were conducted to assess the independent association of each explanatory variable with the practice location. Subsequently, a multivariable (adjusted) analysis was performed to examine the effect of each explanatory variable on the outcome while adjusting for other variables in the model. The exponential estimates from the logistic regression analysis were expressed as odds ratios with 95% confidence intervals. To determine which covariates to include in the regression analyses, we conducted bivariate analyses to examine the associations between each covariate, the exposure variable, and the outcome variable. Covariates that demonstrated a statistically significant association (*p* < 0.05) with the exposure and the outcome variables were considered to exert a confounding effect and were included in the regression model. All covariates in this study, except age, exhibited a statistically significant association with exposure and outcome variables. Although age did not demonstrate a significant association in the bivariate analysis, it was incorporated into the regression model based on robust prior evidence from the literature, underscoring its relevance to the outcome and exposure variables.

## Results

847 students graduated from Dentistry and Oral Health Science courses between 2009 and 2023 at the LRHS. Of these, 819 were matched on the AHPRA register in 2024 and were practicing in Australia. Among the 28 excluded graduates, practice details could not be obtained for 18 graduates from AHPRA. Five graduates practiced internationally, while five did not have home addresses in university records. Of the 819 graduates, 541 (66.1%) were dentists, and 278 (33.9%) were OHTs. More than half of the dentists and OHTs were female, accounting for 56.7% and 81.7% of their respective groups. Most individuals in these professions began their courses between the ages of 16 and 19, with 86.2% belonging to this age group among dentists and 56.5% among OHTs. According to the MMM classification, 11.3% of dentists and 21.6% of OHTs originated from rural/ remote areas, 16.6% and 18% were from regional areas, and 72.1% and 60.4% were from metropolitan areas. After graduation, more than two-thirds, 69.9% of dentists and 70.9% of OHTs, practiced in metropolitan areas, while comparable percentages worked in regional (15.2% and 13.3%) and rural and remote locations (15% and 15.8%) (Table 1).

**Table 1:** Sample distribution according to socio-demographic factors and the practice postcodes of the graduate dentists and oral health therapists

Predictors	Dentists *n*(%)	Oral Health Therapists *n*(%)
**Gender**		
Males	234 (43.3)	51 (18.3)
Females	307 (56.7)	227 (81.7)
**Age at enrolment**		
16–19 years	467 (86.3)	157 (56.5)
*≥* 20 years	74 (13.7)	121 (43.5)
**Graduation year**		
2009–2013	71 (13.1)	69 (24.8)
2014–2018	200 (37)	105 (37.8)
2019–2023	270 (49.9)	104 (37.4)
**Socioeconomic status**		
High	186 (34.5)	49 (17.6)
Medium	256 (47.3)	165 (59.4)
Low	98 (18.2)	64 (23)
**Language spoken within household**		
Non-English speaking	200 (37)	75 (27)
English-speaking	340 (63)	203 (73)
**Home Postcode**		
Rural and Remote	61 (11.3)	60 (21.6)
Regional	90 (16.6)	50 (18)
Metropolitan	390 (72.1)	168 (60.4)
**Practice Postcode**		
Rural and Remote	81 (15)	44 (15.8)
Regional	82 (15.2)	37 (13.3)
Metropolitan	378 (69.9)	197 (70.9)

Table 2 demonstrates a significant relationship between graduates’ practice locations and their places of origin. A significant majority of graduates from metropolitan areas, 79.5% of dentists and 93.4% of OHTs, continued to practice in those areas, with 8.5% and 1.2% moving to regional and 12% and 5.4% relocating to rural/ remote areas, respectively. Among those from regional backgrounds, 40% of dentists and 50% of OHTs returned to practice in regional areas, 47.8% and 44% transitioned to metropolitan regions, and 12.2% and 6% went to rural/ remote locations. Furthermore, 37.7% of dentists and 53.3% of OHTs from rural/ remote origins chose to practice in those areas, with 41% and 30% moving to metropolitan areas and 21.3% and 16.7% relocating to regional areas.

**Table 2:** Bivariate association between graduates’ home location and practice location after graduation

Home location	Practice location		
	Metropolitan	Regional	Rural and remote
Dentists*			
Metropolitan	310 (79.5)	33 (8.5)	47 (12)
Regional	43 (47.8)	36 (40)	11 (12.2)
Rural and remote	25 (41)	13 (21.3)	23 (37.7)
Oral Health Therapists**			
Metropolitan	157 (93.4)	2 (1.2)	9 (5.4)
Regional	22 (44)	25 (50)	3 (6)
Rural and remote	18 (30)	10 (16.7)	32 (53.3)

Chi-square = 92.171 (df = 4), *p* < 0.001*

Chi-square = 168.96 (df = 4), *p* < 0.001**

Tables 3 and 4 present the unadjusted and adjusted multivariable analyses of factors related to graduate dentists’ and OHTs’ place of practice. The multivariable analysis found that age, graduation year, socio-economic status, and household language were not significant factors in choosing regional over metropolitan practice among graduate dentists. In univariate analysis, males were more likely (OR:1.72, 95% CI: 1.06–2.78), and those from high SES were less likely to practice in regional areas (OR:0.37, 95% CI: 0.18–0.78). However, after adjusting for the effect of all other variables, gender and home location were the only predictors associated with practicing regionally. Males had 2.17 times higher odds (95% CI 1.27–3.71), those with a regional origin had 7.72 times higher odds (95% CI 4.17–14.30), and those from a rural or remote background had 4 times higher odds (OR: 3.99, 95% CI 1.70–9.37) of practicing in regional areas compared to their counterparts. When comparing practicing in rural/ remote locations to metropolitan areas in the adjusted analysis, factors associated with choosing rural/ remote practice locations included having a rural/ remote background (OR 7.27, 95% CI 3.36–15.73). Those who graduated between 2009 and 2013 (OR 0.32, 95% CI 0.12–0.82) and 2014–2018 (OR 0.48, 95% CI 0.26–0.85) were less likely to practice in rural/ remote locations than recent graduates (Table 3).

**Table 3:** Univariate and multivariable analysis of the association of the socio-demographic characteristics of the dentists with primary place of practice

	Regional		Rural and remote	
Predictors	Crude odds ratio (95% CI)	Adjusted odds ratio (95% CI)	Crude odds ratio (95% CI)	Adjusted odds ratio (95% CI)
**Gender**				
Males	**1.72 (1.06–2.78)**	**2.17 (1.27–3.71)**	0.67 (0.40–1.11)	0.69 (0.40–1.18)
Females	Ref	Ref	Ref	Ref
**Age**				
16–19 years	0.91 (0.46–1.79)	1.35 (0.63–2.86)	0.99 (0.49- 2)	1.03 (0.49–2.17)
*≥* 20 years	Ref	Ref	Ref	Ref
**Graduation Year**				
2009–2013	1.02 (0.49–2.07)	0.91 (0.41–2.02)	0.50 (0.21–1.18)	**0.32 (0.12–0.82)**
2014–2018	0.88 (0.52–1.49)	0.85 (0.47–1.53)	0.67 (0.40–1.14)	**0.48 (0.26–0.85)**
2019–2023	Ref	Ref	Ref	Ref
**Socioeconomic status**				
High	**0.37 (0.18–0.78)**	0.45 (0.19–1.05)	0.52 (0.25–1.05)	0.92 (0.40–2.11)
Medium	1.01 (0.55–1.88)	0.77 (0.38–1.55)	0.94 (0.50–1.77)	1.34 (0.66–2.74)
Low	Ref	Ref	Ref	Ref
**Language spoken within household**				
Non-English speaking	0.71 (0.42–1.18)	0.91 (0.51–1.63)	0.69 (0.41–1.17)	0.87 (0.49–1.53)
English-speaking	Ref	Ref	Ref	Ref
**Home Postcode**				
Rural and Remote	**4.88 (2.28–10.44)**	**3.99 (1.70–9.37)**	**6.06 (3.18–11.55)**	**7.27 (3.36–15.73)**
Regional	**7.86 (4.44–13.90)**	**7.72 (4.17–14.30)**	1.68 (0.81–3.50)	1.30 (0.59–2.85)
Metropolitan	Ref	Ref	Ref	Ref

*Note*: Significant *p* values are bolded

**Table 4:** Univariate and multivariable analysis of the association between the socio-demographic characteristics of the oral health therapists and their primary place of practice

	Regional		Rural and remote	
Predictors	Crude odds ratio (95% CI)	Adjusted odds ratio (95% CI)	Crude odds ratio (95% CI)	Adjusted odds ratio (95% CI)
**Gender**				
Males	1.23 (0.52–2.92)	2.02 (0.57–7.13)	0.84 (0.34–2.05)	1.76 (0.57–5.41)
Females	Ref	Ref	Ref	Ref
**Age**				
16–19 years	0.69 (0.34–1.39)	0.62 (0.24–1.56)	0.95 (0.49–1.85)	0.72 (0.31–1.66)
*≥* 20 years	Ref	Ref	Ref	Ref
**Graduation Year**				
2009–2013	1.10 (0.44–2.74)	0.68 (0.22–2.13)	1.97 (0.86–4.52)	1.41 (0.50–3.98)
2014–2018	1.02 (0.45–2.29)	1.08 (0.38–3.08)	1.26 (0.57–2.80)	1.34 (0.51–3.57)
2019–2023	Ref	Ref	Ref	Ref
**Socioeconomic status**				
High	0.44 (0.13–1.43)	2.92 (0.41–20.66)	**0.03 (0.01–0.29)**	0.15 (0.01–1.38)
Medium	0.72 (0.30–1.71)	0.73 (0.24–2.16)	**0.29 (0.14–0.60)**	0.47 (0.19–1.15)
Low	Ref	Ref	Ref	Ref
**Language spoken within household**				
Non-English speaking	0.42 (0.16–1.06)	0.44 (0.11–1.74)	**0.41 (0.17–0.97)**	0.69 (0.23–2.06)
English-speaking	Ref	Ref	Ref	Ref
**Home Postcode**				
Rural and Remote	**43.61 (8.85- 214.84)**	**68.68 (10.27–459)**	**31.01 (12.78–75.20)**	**21.52 (8.02–57.75)**
Regional	**89.20 (19.75- 402.91)**	**142.12 (24.2- 834.62)**	2.38 (0.59–9.46)	1.89 (0.46–7.82)
Metropolitan	Ref	Ref	Ref	Ref

*Note*: Significant *p* values are bolded

The multivariable analysis for OHTs revealed that originating from regional, rural/ remote areas significantly influences the choice to practice in regional or rural/ remote areas over metropolitan ones. After adjusting for the effect of other variables, the odds of graduates from regional and rural/ remote areas practicing in regional locations were 142 (OR: 142.12, 95% CI 24.2-834.62) and 69 times (95% CI 10.27–459), respectively. In the adjusted analysis, those from rural/ remote backgrounds were highly likely (OR: 21.52; 95% CI 8.02–57.75) to select rural/ remote practice locations over metropolitan areas (Table 4).

## Discussion

In Australia, most research on tracking practice locations of health students has involved medical students, particularly exploring the factors influencing their decisions to practice in rural and remote areas. These studies consistently show a strong connection between having a rural background and choosing to practice in rural settings [26–29]. However, there is limited evidence on what influences rural practice intentions for dental and oral health professionals in Australia and internationally. Consistent with previous research on medical graduates, we also found that most of our dental and oral health graduates with rural backgrounds took up rural practice. Our findings suggest that for dentists, having a rural background and being male are independent predictors of choosing to practice in rural areas. For OHTs, a rural background remains a significant predictor of rural practice after adjusting for factors such as age at course commencement, graduation year, socio-economic status, and the language spoken at home. Notably, the association of rural background with rural practice appears stronger for OHTs compared to dentists, suggesting that rural-origin OHTs may have a higher likelihood of returning to rural areas to practice than their dentist counterparts.

In 2012, Jones et al. explored the ‘rural background effect,’ investigating which elements of a rural upbringing contribute to the likelihood of becoming a rural doctor. Although the study found no definitive evidence to explain this phenomenon fully, it suggested that environmental factors related to where a person grew up, and their socio-economic background might play a role [30]. The study observed that individuals from lower socio-economic backgrounds were more likely to choose metropolitan practice over rural practice, a contrasting finding with ours. However, a univariate analysis (interactions were not reported) of 569 medical students from the University of Western Australia revealed a strong link between socio-economic disadvantage, rural background, and later rural work, which aligns with our results. The authors proposed that experiencing socio-economic disadvantage in a rural setting might motivate medical students in their career choices after graduation [31].

Exposure to rural settings during training is another important factor influencing whether graduates practice in rural and remote areas. Several studies have examined the impact of undertaking clinical placements in rural locations alongside students’ rural backgrounds on their likelihood of choosing rural practice after graduation. For instance, a multivariate logistic regression analysis of 754 University of Queensland medical graduates found that having a rural background and attending a rural clinical school significantly increased the odds of practising in a rural area by 4.44 times (95% CI 2.38–8.29) for one year of attendance and by 7.09 times (95% CI 3.57–14.10) for two years [29]. Similarly, another study from Western Australia demonstrated that attending a rural clinical school strongly influenced rural practice, particularly for graduates with a rural background. Those graduates with a rural background who participated at the Rural Clinical School of Western Australia (RCSWA) had odds of 7.5 (95% CI 3.5–15.8) of working in rural areas, while the odds dropped to 4.2 (95% CI 1.8–9.2) for rural background graduates who did not attend RCSWA [29]. Overall, the strength of association between home and practice locations observed in our study, was higher compared to past studies.

Our findings also revealed that there were higher odds for practicing in regional and rural/ remote locations among OHTs compared to dental graduates. Several factors could explain this difference. According to Satur et al. (2009), this preference among OHTs may be due to the shortage of dentists in rural areas and the higher demand for restorative services, which allows OHTs to take on more independent roles, including treatment planning and responding to emergencies [32]. Another possible explanation is that regional dentists are more inclined to employ OHTs, driven by increased confidence in their abilities and a better understanding of their full range of clinical skills [33]. Additionally, findings of a descriptive study suggest that factors such as social and community integration, similar earning potential compared to metropolitan areas, and previous rural experience play a significant role in influencing OHTs to practice in rural settings [5].

Although the migration of graduates between practice locations was not explored in this study, there is a possibility that graduates could migrate from regional/ remote to metropolitan locations in their later career stages, which could only be confirmed through longitudinal studies. Several factors noted in the literature might drive this migration, including the appeal of city life, the pursuit of further education or specialised knowledge unavailable in rural areas, and access to metropolitan schooling for children [27, 34]. Conversely, motivations for staying in rural practice include a commitment to patient care, a strong sense of community, the quality and slower pace of rural life, lower living costs, and the unsustainable employment market in metropolitan cities [35–37]. These findings highlight the need for ongoing longitudinal tracking of graduates from both rural and metropolitan programs, similar to that of the Nursing and Allied Health Graduate Outcomes Tracking (NAHGOT) study [38] and the Medical Schools Outcomes Database (MSOD) maintained by Medical Deans Australia and New Zealand [39].

Our study has several strengths and limitations. One of the key strengths of this research is that it represents, to our knowledge, the largest workforce tracking study in dentistry and oral health, focusing on factors influencing rural practice. The large sample size allowed for robust multivariate analyses, examining various factors affecting the likelihood of graduates choosing rural practice. It was conducted at a single, large rural clinical school (LRHS), with clinical placements primarily in regional and rural settings. Additionally, these findings, analysed through the lens of the MMM, are directly relevant to the Australian Rural Health Multidisciplinary Training (RHMT) policy, which targets regions beyond MM1 (metropolitan) areas [27]. However, the study has some limitations, including the reliance on an external source, the existing AHPRA database, although this reduces the risk of self-reporting or recall bias. Additionally, the study could not track those graduates who did not have registration with AHPRA. Another limitation is that these findings may not be generalisable to other regional dental schools. Additionally, our study does not account for the possibility that some participants may have multiple practice locations, which could dilute the FTE spent on rural practice and affect the precision of our analysis of rural workforce outcomes. This limitation highlights the complexity of accurately categorising rural practice engagement and suggests the need for future studies to explore this aspect in greater detail. Lastly, this study relies on area-level SES as a proxy for individual SES. While area-level SES provides valuable contextual insights, it may not fully capture individual-level variations in socio-economic circumstances, which could influence the observed outcomes. Future research should consider incorporating individual-level SES data to enhance the precision of socio-economic analyses.

The Federation of Rural Australian Medical Educators (FRAME) Rural Clinical School Evaluation Data indicates that the strongest predictor of choosing rural practice is the “winning combination” of admitting students from rural backgrounds and offering them rural clinical experiences that nurture and sustain their interest in pursuing rural careers [40]. Our study adds valuable evidence supporting the impact of students’ rural backgrounds in strengthening the Australian rural dental workforce. While graduates with rural origin are more inclined to choose rural practice, it is crucial to acknowledge that this conclusion in our study was drawn from a relatively small percentage of students with regional and rural backgrounds (< 30%). This underscores the need to develop pathways that encourage the recruitment of students with long-term rural residency into dental programs, as outlined in the RHMT program [41]. For this reason, LRHS has developed pathways, such as introducing regional entry codes for admissions, to encourage rural-origin students to enroll in dentistry despite funding challenges to facilitate clinical placements, resulting in 79% of the dentistry student cohort in 2023 being of rural origin. This aligns with the recommendation in the University Accord Final Report to increase university participation rates for regional, rural and remote students from 19.8% up to 24% by 2035 by reducing barriers to study and lifting aspiration [42]. The findings also underscore the importance of increasing government funding to strengthen infrastructure in rural and regional Victoria, which would aid in recruiting students from rural areas, ultimately boosting the rural workforce and improving access to local, timely oral healthcare. Lastly, while our study focuses on the determinants of rural practice among dental professionals, the findings have direct relevance to medical education. Specifically, they underscore the value of rural placements and recruiting students from regional, rural, and remote areas as strategies to enhance the rural workforce, a principle equally applicable to medical students and broader health professional training programs.

## Conclusion

This project aimed to assess how original place of residence by rurality, and other factors influence the likelihood of regional/rural practice among dental and oral health graduates from Australia’s largest rural health school, La Trobe University, between 2009 and 2023. The results indicate that a regional background is the strongest predictor of practicing in regional or rural/ remote locations. Similarly, students from rural and remote locations were highly likely to practice in rural/ remote locations. Notably, the association of rural background with rural practice appears stronger for OHTs compared to dentists. Increasing the recruitment of students with rural backgrounds may positively impact graduates’ decisions to practice in rural areas.

## Data Availability

The data that support the findings of this study are not openly available due to reasons of sensitivity and are available from the corresponding author upon reasonable request, subject to approval from the data custodians at La Trobe University as the data involves personal information of students graduating from La Trobe University.

## Abbreviations

WHO: World Health Organisation
FTE: Full Time Equivalent
NRHA: National Rural Health Alliance
CDBS: Child Dental Benefit Schedule
OHT: Oral Health Therapist
SES: Socioeconomic status
AHPRA: Australian Health Practitioners Regulation Agency
MMM: Modified Monash Model
SEIFA: Socio-Economic Index for Areas – Index of Education and Occupation
OR: Odds Ratio
CI: Confidence Interval
RCSWA: Rural Clinical School of Western Australia
NAHGOT: Nursing and Allied Health Graduate Outcomes Tracking
MSOD: Medical Schools Outcomes Database
RHMT: Rural Health Multidisciplinary Training
FRAME: Federation of Rural Australian Medical Educators
